# Linkage and association analysis in pedigrees from different populations

**DOI:** 10.1186/1471-2156-6-S1-S59

**Published:** 2005-12-30

**Authors:** Joseph Beyene, Jun Yan, Celia MT Greenwood

**Affiliations:** 1 Program in Population Health Sciences, Research Institute, Hospital for Sick Children, 555 University Avenue, Toronto, Ontario, M5G 1X8, Canada; 2 Program in Genetics and Genomic Biology, Research Institute, Hospital for Sick Children, 555 University Avenue, Toronto, Ontario, M5G 1X8, Canada; 3 Department of Public Health Sciences, University of Toronto, Toronto, Ontario, Canada

## Abstract

Using the Genetic Analysis Workshop 14 simulated datasets we carried out nonparametric linkage analyses and applied a log-linear method for analysis of case-parent-triad data with stratification on parental mating type. We proposed and applied a random effect modelling approach to explore the impact of population heterogeneity on tests of association between genetic markers and disease status. The estimated genetic effect may appear to be strongly significant in one population but nonsignificant in another population, leading to confusion about interpretation. However, when results are interpreted in the light of a random effects model, both studies may be making similar statements about a genetic effect that varies depending on environment and background.

## Background

It has proven to be very difficult to validate linkage and association findings for complex diseases. Part of the reason for the inconsistency may be due to real differences in effect sizes across populations and studies. Methods that explicitly model potential heterogeneity across populations are useful in clarifying reasons behind this variability as well as in estimating model parameters accurately. The objective of our study is to use the Genetic Analysis Workshop 14 (GAW14) simulated datasets to explore the impact of population heterogeneity on tests of association between genetic markers and disease status, and to use random effects models to account for this heterogeneity. We were also interested in estimating gene × covariate interactions and heterogeneity associated with these effects. We adopted a three-stage analytical approach without knowledge of the generating model: 1) linkage analysis to find "interesting" regions, 2) association analysis for selected markers in separate populations, and 3) use of random effects models to combine the association test results across populations and to examine heterogeneity.

## Methods

We conducted a combined linkage and association analysis using GAW14 simulated datasets. The simulated data consists of phenotypic as well as genotypic information from four populations: Aipotu, Danacaa, Karangar, and New York City for the study of Kofendred personality disorder (KPD). Twelve disease symptom traits were also included. Microsatellite and single-nucleotide polymorphism (SNP) markers were available for 10 chromosomes.

We first carried out a nonparametric linkage (NPL) analysis [1] within each population for the first replicate, using the given affection status. Both the microsatellite and SNP marker data for the 10 chromosomes were used in the linkage analysis. We then repeated these analyses in an additional two randomly selected replicates (replicates 11 and 78) from each population to assess consistency of results.

We used a log-linear model for estimating association that was designed for the analysis of case-parent-trios [2,3], and estimates log-relative risk parameters for a single variant allele. Using appropriate parameterizations allows modelling of different modes of transmission (additive, dominant, or recessive). Likelihood-ratio tests of linkage can also be obtained. The model includes six intercept terms that estimate baseline disease risk by parental mating type, where mating type is defined by the configuration of risk alleles in the parents. This stratification on parental mating types protects against bias in the estimates of the genetic-association parameters due to population stratification. The model can be extended to include gene × covariate interactions. It should be noted that the estimates of the main covariate effect are not identifiable. The model was implemented in the SAS statistical software, version 8.02 (SAS Institute Inc., Cary, NC).

Based on the linkage analysis results, we selected a small number of interesting regions for association mapping using "purchased" fine-mapping markers. From each population, we selected 300 independent case-parent trios. One affected child was randomly selected from each pedigree, with his/her parents. For populations Aipotu, Danacaa, and Karangar, replicates 1, 11, and 87 were used. For New York, where replicates contained only 50 pedigrees, trios were also selected from replicates 2, 3, and 4. Association analysis was carried out for selected markers with covariates based on gender and the anxiety-related sub-phenotype.

A generalized linear mixed modelling (GLMM) framework was used to explore variability across populations and to combine risk estimates [4]. This framework is an extension of the normal mixed model that accommodates non-normal error distributions. In our case, the genetic-association parameters were assumed to be normally distributed across populations, while the six intercept terms were held fixed. Conditional on the random effects, the count outcome has a Poisson distribution with conditional mean *μ *= *E*(*Y*|*b*) = *h*(*η*) with *η *= *X β *+ *Zb*, where h is the logarithmic link function, *X *is a design matrix corresponding to the fixed-effect parameters *β*, and Z is a design matrix associated with the random-effect parameters b. Detailed notations and formulation of the basic Poisson model of Weinberg are given elsewhere [2,3]. The resulting Poisson generalized linear mixed model was fitted using a SAS macro called GLIMMIX [5].

All analyses were conducted without knowledge of the generating model.

## Results

The NPL analyses using data from replicate 1 showed that, of the 10 chromosomes, only chromosomes 1, 3, 5, and 9 contained statistically significant regions, in at least one of the four populations (Table 1). For the Danacaa population, strong evidence for linkage was found on chromosome 1 from both the microsatellite (marker: D01S0023; NPL score = 4.48) as well as SNP (marker: C01R0052; NPL score = 5.19) data. We found regions on chromosome 3 with strong linkage signals in three populations (Aipotu, Karangar, and New York). For the Karangar population, the strongest signals were observed on chromosomes 5 and 9 (both for microsatellite and SNP). After repeating the NPL analyses with two other randomly selected replicates (replicates 11 and 87), we found consistent significant findings on chromosomes 1, 3, 5, and 9.

Fine mapping packages were purchased for chromosome 3 and 5 near the linkage peaks. We chose these regions in order to contrast linkage findings that appeared consistent across populations (chromosome 3) with a region demonstrating variability in the strength of the linkage evidence across populations (chromosome 5); we hoped that the generating models for this chromosome 5 region might vary across populations. We obtained packages 152 and 153 for chromosome 3 and packages 207–211 for chromosome 5.

We did not find significant allelic association for markers on chromosome 5, and therefore no results are reported. For chromosome 3, Table 2 summarizes results from the log-linear modelling of selected markers. An additive genetic model was assumed. One of the markers (B03T3056) shows significant association with disease as well as gene × environment interaction with the anxiety-related covariate. The association results appear consistent across the four populations, but the magnitude of the gene × environment interaction varied significantly. An adjacent marker (B03T3057) also showed a significant association (for all populations) and gene × environment interaction for only 2 of the four populations. Likewise the next SNP, B03T3058, showed significant interaction with the binary covariate (anxiety-related symptoms) for three of the populations (with the exception of Aipotu). Thus in some populations the disease-marker associations in the affected children with anxiety-related symptoms are significantly different from the associations in those without the symptoms. We also included sex as a covariate, but we did not find any significant interactions.

SNPs B03T3057 and B03T3056 are used to illustrate the results from the mixed model where we assumed that the allelic and interaction risk estimates, under an additive genetic model, were random across populations (a GLMM framework). After combining across populations, the *p*-value for marker B03T3057 for the gene × anxiety interaction was less striking but still significant (estimate = 0.24; SE of estimate = 0.06; *p*-value = 0.0345) while the *p*-value for the main effect of testing association with the variant allele increased to *p *= 0.0170 (estimate = 0.36; SE of estimate = 0.07). The random-effect variance components are not significantly different from zero.

For the adjacent marker B03T3056, the gene × anxiety interaction effect appeared non-significant (estimate = 0.19, SE of estimate = 0.07; *p *= 0.0791); however, the main effect remained significant (estimate = 0.67; SE of estimate = 0.08; *p *= 0.0043). In this model the random effect variance components are significantly different from zero.

## Discussion

Valid and powerful statistical methods are useful in the discovery of genes involved in disease susceptibility and detection of gene × environment interactions. We have conducted a combined linkage and association analyses using the GAW 14 simulated datasets. We identified several interesting regions using linkage analysis and further investigated some of the regions with fine mapping approaches. Our results suggest that there may be significant variation in the four populations, especially in gene × environment interactions. Our analyses were performed without knowledge of the generating model.

We adopted a log-linear model to investigate allelic associations for selected markers. This modelling approach can be extended to include parameters for several desirable quantities such as imprinting effects and gene-environment interactions. As a member of the family of generalized linear models, the usual optimal asymptotic properties apply and it can be implemented using widely available statistical packages. This approach can also be regarded as a generalization of the approach proposed by Schaid and Sommer [6], as a maximum-likelihood method conditional on parental genotypes.

In the presence of gene × covariate interactions, the log-linear model must include separate intercept terms for each level of the covariate in order to ensure protection against hidden population stratification. However, for small datasets, the counts in the contingency table become sparse and it can become difficult to estimate all the required parameters (12 intercept parameters for one binary covariate). Therefore, a trade-off becomes necessary between full-immunity to population stratification and reliable estimates. All models fitted here used only one set of intercept parameters (6 intercepts). These models will give unbiased estimates of the genetic associations if either the covariate frequency (gender or anxiety) does not vary across any hidden population substructure, or the covariate is not associated with the genetic effect. In the GAW simulation, there was no hidden population substructure within each of the four stated populations, and therefore the more parsimonious models were appropriate.

Validation of association studies is a continuing problem, and part of the difficulty is attached to inadequate power in the various studies, as well as, of course, genetic model heterogeneity in different populations or samples. By estimating genetic risk parameters using the Weinberg model, we can see whether the estimates of genetic risk are similar in different populations, rather than just comparing parameters. The Poisson GLMM approach we applied here allows exploring differences across populations (variability in risk estimates) as well as combining estimates meta-analytically. The estimated genetic effect may appear to be strongly significant in one population but non-significant in another population, leading to confusion about interpretation. However, when results are interpreted in the light of a random effects model, both studies may be making similar statements about a genetic effect that varies depending on environment and background.

The estimates of association from the GLMM were smaller than from the separate Poisson models. This may be due to inflation in the estimates of the association parameters from the individual populations due to small sample bias, or to parameter shrinkage associated with the incorporation of extra sources of variability. Further investigation of this effect is warranted.

Our approach of combining results across populations is an implementation of a meta-analysis strategy to understand and summarize results across independent studies. Meta-analysis can also be used to identify factors that may explain heterogeneity. Such an approach may prove useful in genetic studies where results vary across populations. A further extension to our mixed model approach may be developed in a fully Bayesian framework.

## Conclusion

We identified several regions showing evidence for linkage and association in the GAW14 simulated data. We also proposed a strategy for examining heterogeneity of association test results by using models that can include covariates, and implemented mixed models to allow for genetic effects to vary across populations. Although there is still a long way to go to dissect the genetics of complex diseases, data integration approaches such as our multi-level modelling framework might help elucidate genetic and environmental contributions to the risk of diseases.

## Abbreviations

GAW: Genetic Analysis Workshop

GLMM: Generalized linear mixed model

KDP: Kofendred personality disorder

NPL: Nonparametric linkage

SNP: Single-nucleotide polymorphism

## Authors' contributions

JY carried out the linkage and log-linear association analyses. JB wrote the manuscript and carried out the mixed model analyses, using ideas developed by himself together with CMTG. CMTG assisted in editing the manuscript.

## Figures and Tables

**Table 1:** Peak NPL score along with peak locations for chromosomes with suggestive and/or strong linkage signals by population and marker type (Microsatellite versus SNP)

		Microsatellite Map			SNP Map		
			
Chromosome	Population	Marker*	NPL	Location†	Marker*	NPL	Location†
1	New York	S0026	2.878	187.5	R0055	2.746	162
	Danacaa	S0023	4.484	165	R0052	5.190	153
	Aipotu	S0010	2.566	67.5	R0023	2.472	66
	Karangar	S0023	1.689	165	R0050	1.810	147
3	New York	S0127	4.523	307.5	R0280	3.217	276
	Danacaa	S0127	2.947	307.5	R0280	2.177	276
	Aipotu	S0127	4.362	307.5	R0279	3.763	273
	Karangar	S0127	3.998	307.5	R0280	4.682	276
5	New York	S0176	3.479	30	R0387	3.218	27
	Danacaa	S0180	1.710	60	R0455	1.905	231
	Aipotu	S0173	1.957	7.5	R0380	1.329	6
	Karangar	S0172	4.587	0	R0380	4.829	6
9	New York	S0368	1.687	157.5	R0812	1.176	147
	Danacaa	S0372	3.090	187.5	R0820	2.234	171
	Aipotu	S0364	1.565	127.5	R0832, R0856	1.414	207, 279
	Karangar	S0347	4.583	0	R0764	4.283	3

*Marker names have been shortened to save space (e.g., S0026 stands for D01S0026 and R0055 is C01R0055, where in this case 01 indicates the markers are on chromosome 1).

^†^Peak locations are in centiMorgans.

**Table 2:** Log-linear model results for four adjacent markers on chromosome 3 (package 152) for different populations

		LD without covariates		G × E interaction, anxiety as a covariate	
			
Marker	Population	Estimate (SE)	*p*-value	Estimate (SE)	*p*-value
B03T3055	Aipotu	0.0940 (0.1160)	0.4175	- 0.0137 (0.0827)	0.8687
	Karangar	-0.1552 (0.1164)	0.1824	0.2440 (0.0873)	0.0052
	Danacaa	-0.0261 (0.1143)	0.8191	0.0962 (0.0862)	0.2645
	New York	0.2513 (0.1222)	0.0398	0.2315 (0.0819)	0.0047

B03T3056	Aipotu	0.799 (0.1242)	<0.0001	0.0266 (0.0730)	0.7152
	Karangar	0.7584 (0.1284)	<0.0001	0.2658 (0.0755)	0.0004
	Danacaa	0.8183 (0.1265)	<0.0001	0.1624 (0.0741)	0.0285
	New York	0.7233 (0.1231)	<0.0001	0.3222 (0.0761)	<0.0001

B03T3057	Aipotu	0.4773 (0.1254)	<0.0001	0.0769 (0.1049)	0.4637
	Karangar	0.4675 (0.1282)	0.0003	0.3937 (0.1171)	0.0008
	Danacaa	0.6061 (0.1196)	<0.0001	0.1543 (0.1019)	0.1302
	New York	0.4317 (0.1208)	0.0004	0.3608 (0.1037)	0.0005

B03T3058	Aipotu	0.4282 (0.1193)	0.0003	0.0093 (0.0789)	0.9058
	Karangar	0.5460 (0.1221)	<0.0001	0.2114 (0.0787)	0.0072
	Danacaa	0.6826 (0.1259)	<0.0001	0.1728 (0.0753)	0.0217
	New York	0.3452 (0.1224)	0.0048	0.3154 (0.0793)	<0.0001
